# Bayes Factor-Based Regulatory Gene Network Analysis of Genome-Wide Association Study of Economic Traits in a Purebred Swine Population

**DOI:** 10.3390/genes10040293

**Published:** 2019-04-10

**Authors:** Jungjae Lee, Ji-Hoon Kang, Jun-Mo Kim

**Affiliations:** 1 Jung P&C Institute, Inc., 1504 U-TOWER, Yongin-si, Gyeonggi-do 16950, Korea; jungjae.ansc@gmail.com; 2 Research & Development Center, PatentPia Inc., Seoul 06223, Korea; dvdvkj@gmail.com; 3 Department of Animal Science and Technology, Chung-Ang University, Anseong-si, Gyeonggi-do 17546, Korea

**Keywords:** Association weight matrix, Bayes factor, economic trait, single nucleotide polymorphism

## Abstract

Early stage prediction of economic trait performance is important and directly linked to profitability of farm pig production. Genome-wide association study (GWAS) has been applied to find causative genomic regions of traits. This study established a regulatory gene network using GWAS for critical economic pig characteristics, centered on easily measurable body fat thickness in live animals. We genotyped 2,681 pigs using Illumina Porcine SNP60, followed by GWAS to calculate Bayes factors for 47,697 single nucleotide polymorphisms (SNPs) of seven traits. Using this information, SNPs were annotated with specific genes near genome locations to establish the association weight matrix. The entire network consisted of 226 nodes and 6,921 significant edges. For in silico validation of their interactions, we conducted regulatory sequence analysis of predicted target genes of transcription factors (TFs). Three key regulatory TFs were identified to guarantee maximum coverage: AT-rich interaction domain 3B (ARID3B), glial cell missing homolog 1 (GCM1), and GLI family zinc finger 2 (GLI2). We identified numerous genes targeted by ARID3B, associated with cellular processes. GCM1 and GLI2 were involved in developmental processes, and their shared target genes regulated multicellular organismal process. This system biology-based function analysis might contribute to enhancing understanding of economic pig traits.

## 1. Introduction

Growth rate traits, such as average daily gain (ADG) and days to 90-kg body weight (DAYS), and production traits, such as backfat thickness (BFAT) and lean percent (PCL), have been typically considered as important traits, as they play a major role in the economic success of Korean pig breeding programs. Moreover, the lifetime total number born (LTTNB), lifetime number born alive (LTNBA), and weaning to estrus interval (WEI) are also economically important for sow longevity and reproduction. To date, these economic traits have been genetically improved successfully based on traditional best linear unbiased prediction (BLUP), and breeding values of economic traits have been used with a selection index to select elite lines in Korean pig breeding.

Recently, genomic information in the form of dense single nucleotide polymorphism (SNP) marker panels (e.g., Illumina, Neogen-GeneSeek, and Affymetrix) has become available for genetic evaluation, owing to improvements in genotyping technology and statistical methods. One of its applications is in genome-wide association study (GWAS), which has become a powerful genomics tool to identify genetic loci or genes underlying quantitative traits in domestic animals [1]. The single marker regression (SMR) method was first introduced in GWAS. However, the original and other modified SMR versions might have limited application in the estimation of SNP marker effect in the entire genome owing to various reasons: the SMR methods tend to overestimate the SNP marker effect as they ignore the effects of other SNP markers [2] and they are insufficient to detect SNPs with small effects. Therefore, it is useful to apply the Bayesian approach, which fits all possible multiple markers simultaneously, and was originally developed for genomic selection [3]. It has been shown to be a better approach for quantitative trait loci (QTL) mapping or GWAS than the SMR method in detection power [2,4,5]. Furthermore, Fortes, et al. [6] recently suggested a system biology-based strategy called association weight matrix (AWM) that integrates information from GWAS to study complex traits and identify candidate genes. Several researchers have applied this methodology using *p*-values of the GWAS result, but there has been no report of the use of a combination of AWM-methodology and Bayesian approach thus far [7,8,9,10].

The objectives of the present study were to: (i) conduct a GWAS using the Bayesian method to investigate the genetic architecture and chromosomal regions associated with economic traits of pigs, including growth rate and production-related traits such as litter size information in Yorkshire population using dense SNP panels and (ii) identify the co-associated regulatory network of the multi-trait Bayesian approach GWAS using the AWM methodology.

## 2. Materials and Methods 

### 2.1. Genotypes and Phenotypes

From 2014 to 2017, 2681 Yorkshire pigs were genotyped using Illumina PorcineSNP60 version 2 (Illumina, Inc., San Diego, CA) comprising 61,565 SNP markers. After excluding SNPs that were unmapped, on sex chromosomes, and those with poor call rates (<0.95), the available number of SNP markers was 47,697. Duplicated animals (n = 60) caused by re-genotyping to obtain acceptable call rates, and animals with lower call rates (n = 30) were removed after comparing their call rates. We also removed animals (n = 19) with call rates <0.90. The parentage test was performed using SEEKPARENTF90 software (INIA, Las Brujas, UY) [11] with known parent-offspring in the pedigree file. A conflict threshold of 10% was used to detect paternity error and correct the pedigree file. Consequently, 244 genotyped animals were removed, and the pedigree file was corrected. Furthermore, genotype identification data that could not be matched to the corresponding animals in the phenotypic and pedigree files were removed, leaving 1833 animals for further GWAS. Missing SNP genotypes (0.27%) were imputed using FImpute version 2.2 [12].

All experimental procedures involving animals were conducted in accordance with the Guide for Care and Use of Animals in Research and approved by the Institutional Animal Care and Use Committee of the National Institute of Animal Science (No. 2015-137).

### 2.2. Measurement of Economic Traits

Body weights were measured once during performance testing (at approximately 150 days). The ADG was calculated as the difference in final weight and initial weight divided by the number of days at the time of performance testing. The DAYS was estimated according to the recommendations of the Korean Swine Performance Recording Standards (KSPRS), adjusted from birth to the time of performance testing. The BFAT was calculated based on the average fat thickness values of the shoulder (on the fourth thoracic vertebrae), mid-back (on the last thoracic vertebrae), and loin (on the last lumbar vertebrae) measured using the A-mode (amplitude mode) ultrasound device (PIGLOG 105). The PCL was calculated according to the recommendations of the KSPRS, following previously reported procedures [13]. More details of the correction formula for growth and production traits were reported by Choy et al. [14]. The three-sow reproduction-related traits LTTNB, LTNBA, and WEI were obtained from real phenotypic records.

### 2.3. Response Variable

Phenotypic data of 39,518 purebred Yorkshire pigs were collected from three Korea GGP farms between 2012 and 2017. Pedigree data from 99,694 individuals were also used. Table 1 shows the number of available records, phenotypic means and their standard deviation, variance component, and heritability for each trait. Genetic parameters, breeding values, and the corresponding reliability were estimated using a pedigree relationship matrix fitted with ASReml version 4.1 software (VSN International Ltd., Hemel Hampstead, UK) [15] for growth rate (ADG and DAYS), production traits (BFAT and PCL), and reproductive traits (LTTNB, LTNBA, and WEI). A multi-trait animal model was used for those parameters and estimated breeding value (EBV) including fixed effects of farm, birth-year, season, and sex. Further, the deregressed estimated breeding value (DEBV) was adjusted for parental information by a combination of deregression after adjusting for parental average such that the DEBV information contained only their phenotypic information and that of their descendants. The response variable was weighted to account for the heterogeneous variance of DEBV due to the differences in EBV reliabilities among the genotyped animals. The weighting factor [16] for animal i ($w_{i}$) was calculated as follows:

$$w_{i}=\;\frac{\left(1-h^{2}\right)}{\left\{c+\left[\left(1-\;r_{i}^{2}\right)/r_{i}^{2}\right]\right\}h^{2}}$$

where, $r_{i}^{2}$ is the reliability of DEBV, $h^{2}$ is the heritability of the trait, and c is the proportion of genetic variation that could not be explained by markers. In the present study, c was assumed to be equal to 0.40 [17]. After removing animals with reliability <0.10, 1596 registered Yorkshire pigs were used in the GWAS.

### 2.4. Bayesian Method for Genome Wide Association Study 

The BayesB [3] method with π set to 0.99 and weighting factors was used to estimate the effect of SNP markers and calculate variances attributed to every non overlapping 1-Mb genome window using GenSel4R software [18]. BayesB method uses a mixture model that assumes some fraction π of SNP markers have zero effect and every SNP marker has locus-specific variances. For each trait, the following model was fitted to estimate marker effects:

$$y_{i}=\mu +\;\sum_{j=1}^{k}Z_{ij}u_{j}\delta_{j}+e_{i}$$

where, $y_{i}$ is response variable (DEBV) of animal i for the respective trait; μ is the population mean; k is the number of markers; $Z_{ij}$ is allelic state at locus j in individual i; $u_{j}$ is the random substitution effect for marker j, which follows a mixture distribution for this random substitution effect according to indicator variable ($\delta_{j})$, a random 0/1 variable indicating the absence or presence of marker j in the model, with $u_{j}$ assumed normally distributed N(0,$\sigma_{u}^{2}$) when $\delta_{j}=1$; and $e_{i}$ is a random residual effect assumed to be normally distributed N(0,$\;\sigma_{e}^{2}$). The posterior distribution of the parameters and effects was obtained using Gibbs sampling for 110,000 Markov chain Monte Carlo (MCMC) iterations, of which the first 10,000 were discarded for burn-in before estimating posterior means of marker effects and variances, saving the results every five cycles. The accumulated frequency across iterations of the chain for a particular SNP based on prior π fitted in the model (referred to as “model frequency”) can be used as evidence of an informative SNP or QTL [19]. However, the adjacent SNPs might be in high linkage disequilibrium (LD) with the same QTL in a high-density SNP panel and, hence, the effect of QTL and the SNP model frequency would be spread over all SNPs in high LD, which can result in the underestimation of individual SNP effect and model frequency [20]. Therefore, a window approach, which accumulates the effects of adjacent SNPs for each 1-Mb region, has been implemented in GenSel4R software, and this 1-Mb window approach was used to identify informative genomic regions accounting for LD. Initial values for genetic and residual variances for BayesB were estimated using a linear mixed model implemented in ASREML (Table 1). All procedures were performed using GenSel4R software [18]. In total, 2452 consecutive non-overlapping 1-Mb windows across the whole genome were included in the GWAS. 

### 2.5. Identification of Significant Window Regions and Single Nucleotide Polymorphism Markers

An additive genetic variance of 1.0%, which was estimated as a fraction of the total genetic variance explained by all SNPs, was used as the significance level of putative informative 1-Mb window region. Unlike the single marker regression approach, there is no P-value for significance of SNP marker in Bayesian approaches. Therefore, the posterior probability of inclusion of each SNP marker into the model (model frequency) in MCMC cycles is mostly used as a criterion for detecting QTLs [21]. Bayes factor (BF) derived from model frequency was used to determine the SNP with a significant association within this region.

$$\mathrm{BF}=\frac{\hat{p_{i}}/\left(1-\hat{p_{i}}\right)}{\left(1-\pi \right)/\pi }$$

where, π is the prior probability and $\hat{p_{i}}$ is the posterior probability of the fraction of times the SNP was distributed. Following the definitions of Kass et al. [22] for the strength of an association based on their range of values, the SNP markers with BF > 3.2, > 20, and > 100 were considered “suggestive,” “strong,”, and “decisive” evidence, respectively.

### 2.6. Association Weight Matrix Construction

The AWM consists of rows representing genes and columns representing the additive effect of each trait based on the results of the GWAS [6]. Before construction, we selected a “weakly” significant criterion of Bayes factor of ≥ 3.2 [22], and BFAT was used as a key phenotype among the seven traits. Firstly, SNPs that were significantly associated with BFAT or associated with at least two phenotypes were selected. Secondly, the SNPs satisfying the distance information of SNPs to the nearest annotated coding region of the gene were additionally filtered, i.e., those that were either <2500 bp or >1.5 Mb away from the nearest gene were eliminated. Finally, only one SNP was selected to represent the gene (the first criterion was the number of statistically significant traits to the SNP and the second was more significant to the key phenotype). The partial correlation and information theory (PCIT) algorithm was used to identify a significant interaction among the genes and SLP-related traits using the PCIT library in R [23]. The hierarchical clustering option in PermutMatrix software [24] was used to visualize the AWM. To visualize the network of the AWM genes, every significant co-associated gene was applied in Cytoscape, and the network density of each gene was obtained using the MCODE sub-package [25].

### 2.7. Network Analysis Using Transcription Factor and Target Gene Information

To provide in silico validation of the gene-gene interactions and validate the whole network, among various available methods, we used bio-informatics analysis that predicts TFs and their target genes [6]. To determine whether a gene is a TF or not, it was compared with the pig and human transcription factor database list sets [26]. The genes identified as TFs required motif information. *Sus scrofa* motif information from the CisBP database [27] was mainly used, and *vertebrate* data from JASPAR [28] were used to supplement missing information. We extracted the flank region sequence (upper 2000 bp) of every gene in the whole network from the Ensembl BioMart database [29]. To identify locally overrepresented TF binding sites (TFBS), the FIMO tool [30] was used. It detected all the TFBS and extracted the significant clusters (*P* < 0.001) by calculating their score functions [31]. The top three TFs were chosen to satisfy the maximum coverage as previously reported [7]. The classification analysis of the function of node gene was analyzed by inputting the list of gene ensemble ID into the Panther classification system [32]. 

## 3. Results and Discussion

### 3.1. Genome-Wide Association Study Using Single Nucleotide Polymorphisms Markers with Illumina PorcineSNP60

**Figure 1:**
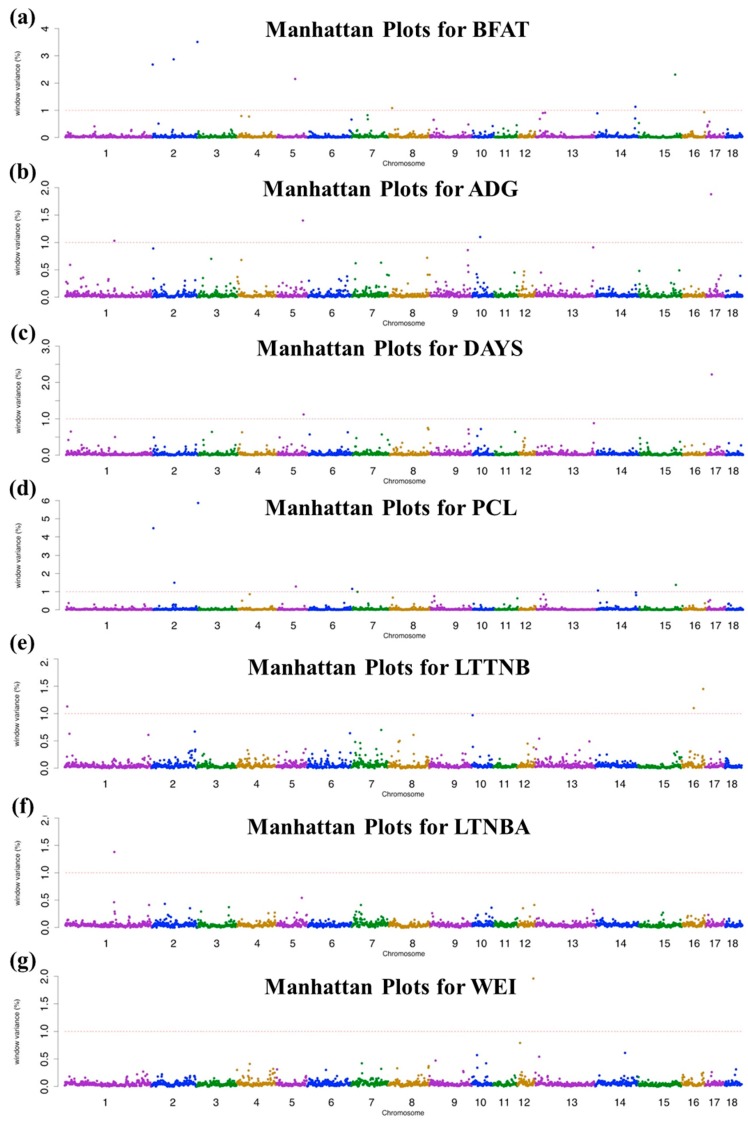
Manhattan plot of genome-wide association study result of 18 porcine autosomes. The y-axis indicates window variance (%) and x-axis represents the pig autosomal chromosome physical map. The red dot horizontal lines represent the threshold of the percent variance of 1-Mb genomic region used was above 1.0% to identify associations with traits: (**a**) backfat thickness (BFAT), (**b**) average daily gain (ADG), (**c**) days to 90-kg body weight (DAYS), (**d**) lean percent (PCL), (**e**) lifetime total number of born (LTTNB), (**f**) lifetime number of born alive (LTNBA), and (**g**) weaning to estrous interval (WEI).

We performed a GWAS using SNP markers on Illumina PorcineSNP60 based on several parameters estimated by the BayesB method (i.e., the absolute SNP marker effect, model frequency, and the genetic variances explained by SNP markers). Bayesian GWAS applies the threshold for the significance of SNP markers based on the derivative of model frequency (i.e., BF) [21]. However, a single QTL could spread the effects over multiple SNPs when using high-density SNP panel as a high linkage disequilibrium (LD) within adjacent SNP markers. These results may lead to an increase in the probability of false positives and false negatives [19]. To overcome these problems, we used two thresholds: (i) additive genetic variance by accumulating within 1-Mb chromosomal regions and (ii) BF based on the model frequency. The results of the GWAS of growth, productive, and reproductive traits including chromosomal and window location (Mb), the percentage variance of 1-Mb genome windows, SNP, physical genome position (Mb), additive effect of the significant SNP marker within these regions, and BFs in Yorkshire pigs are presented in Table 2 and Table 3. In this study, the threshold of percentage variance of 1-Mb genomic region and BF used to identify associations with traits were > 1.0% and 20, respectively. The Manhattan plots for the analyzed traits are shown in Figure 1. 

### 3.2. Growth-Related Traits

In the present study, the most informative 1-Mb window region was detected on SSC17 at 17 Mb, which explained 1.88% and 2.22% of additive genetic variances for ADG and DAYS traits, respectively. Furthermore, the most significant QTL was found at 17.55 Mb on SSC17 (rs342665431) with the highest BF: 298.75 and 764.87 for ADG and DAYS traits, respectively. A previous study [20] reported that the most significant SNP (rs342665431) was from the *BMP2* gene on SSC17, which is consistent with our results. The *BMP2* gene is a member of the bone morphogenetic protein family that regulates early myogenesis. We also found identical informative 1-Mb window regions between the growth-related traits and SNP located at the 93-Mb position of SSC5 (rs345168974) with 1.40% and 1.12% additive genetic variances for ADG and DAYS traits, respectively.

### 3.3. Production-Related Traits

We found 15 significant QTLs within 12 informative chromosomal regions (significance level > 1.0% additive genetic variance or BF > 20) on SSCs 2, 4, 5, 6, 7, 8, 14, 15, and 16 for BFAT and PCL. The most significant 1-Mb window region explaining 3.51% and 5.87% of additive genetic variances was captured on SSC2 at 162 Mb, including two SNPs (rs81341288 and rs81328276) in BFAT and PCL. The QTL window located on SSC at the beginning, which explained 2.68% and 4.48% of additive genetic variances, included SNPs (rs81317307 and rs81318741) for BFAT and PCL. Furthermore, rs81317307 was the most significant SNP based on BF (1268.40) associated with PCL. Van Laere, et al. [33] reported that the *IGF2* gene on SSC2 has an important role in the development of skeletal muscle and BFAT as well as postnatal muscle regeneration and hypertrophy. Other QTL windows were also detected on SSC2 at 76 Mb, which explained 2.87% and 1.49% of additive genetic variances for two production-related traits. The most significant SNP located on SSC5 at 65 Mb (rs81343150) was identified based on BF (304.09) for BFAT. 

### 3.4. Reproduction-Related Traits

Some candidate chromosomal regions and QTLs associated with reproduction-related traits were identified. We found six significant QTLs within five informative chromosomal regions on SSCs 1, 12, and 16 for reproduction-related traits. Among those genes, the superoxide dismutase 2 (*SOD2*) gene has been reported to have one polymorphism associated with male infertility [34]. Considering the results of the GWAS, a few QTLs for LTTNB, LTNBA, and WEI but no QTL were identified for NPW, which might be due to low heritability (Table 1) and relatively smaller sample size for detecting significant QTL regions. Another reason for this result might be high criteria of significance. Because of the high cut-off criteria for the significance level of the traditional single trait GWAS strategy, it is difficult to determine the useful QTL on those reproduction-related traits [35].

### 3.5. Co-Association Network Based on Association Weight Matrix

**Figure 2:**
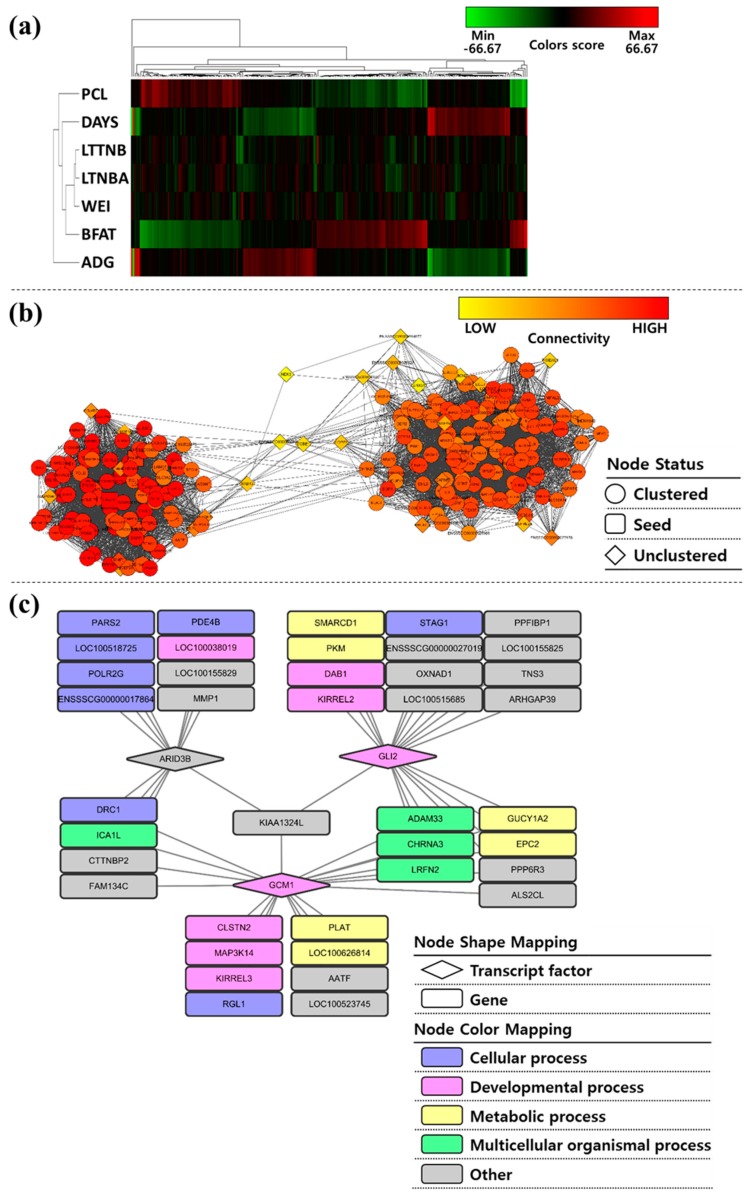
Functional gene network from the genome-wide association study using association weight matrix (AWM). (**a**) Visualizing the AWM using permutmatrix software. Each cell (*i, j*) is the z-score normalized additive effect of *i*th-trait on *j*th-SNP. (**b**) Entire network: The nodes represent 226 genes and the 6,921 edges represent significant correlations between the nodes. The color scale corresponds to the MCODE score, where the red nodes represent a high network density. (**c**) A subset of the network showing the top three transcription factors in the in silico validated targets. The diamond-shaped nodes are transcription factors.

The constructed AMW consisted of 215 (211 genes and 4 SNPs) × 7 (traits), and each cell represents their *z*-value normalized additive effect (Figure 2a). To visualize this, Permutmatrix software [24] was used. There were three main obvious findings in the visualized matrix. First, the genes had strong effects on both of body-related traits (PCL and BFAT) and growth-related traits (DAYS and ADG). Second, PCL and BFAT, and DAYS and ADG pairs, respectively, were almost the compensate tendency by the effects of the genes. This was an obviously understandable deduction from the meaning of the traits (e.g., meat percentage information versus fat information for PCL and BFAT). Another finding is that it was difficult to identify the specific tendency of the effect on reproductive traits (LTTNB, LTNBA, and WEI) because it was quite different from that on the traits of the two groups mentioned above and it had a weak effect (relatively dark compared to other groups). Therefore, research on those reproduction-related traits is limited by the traditional GWAS method [35] and AWM-based approach has recently emerged as a useful option [6,10]. 

The whole network created by the PCIT analysis based on AWM information consists of 226 nodes and 6921 edges (Figure 2b). The rectangle node indicates seed on the network and the diamond node is uncluttered based on the MCODE analysis of Cytopscape application. Based on the comparison with the TF database, ARID3B, ATF6B, DMTF1, GCM1, GLI2, ISL1, KDM5B, KLF17, NFYC, NPAS3, and WDHD1 were identified as TFs. Among them, five TFs (ARID3B, GCM1, GLI2, ISL1, and NFYC) had motif information. Each TF and its target gene network were matched using FIMO tool analysis and, finally, the top trio network consisting of 43 genes and centered on ARID3B, GCM1, and GLI2 was constructed (Figure 2c). 

The AT-rich interaction domain 3B (ARID3B) encodes a member of the AT-rich interaction domain (ARID) family of DNA-binding proteins [36]. Studies have reported that the *ARID3B* gene affects the regulation of limb development [37]. However, the function of the *ARID3B* gene in pigs has not been studied comprehensively. The ARID3B has 13 in silico validated target genes (*CTTNBP2*, *DRC1*, *ENSSSCG00000017864*, *FAM134C*, *ICA1L*, *KIAA1324L*, *LOC100038019*, *LOC100155829*, *LOC100518725*, *MMP1*, *PARS2*, *PDE4B*, and *POLR2G*), most of which (six out of 13 genes, *DRC1*, *ENSSSCG00000017864*, *LOC100518725*, *PARS2*, *PDE4B*, and *POLR2G*) are involved in the cellular process (Figure 2c). Many of them were essential genes for survival. For example, *DRC1* is essential for motile cilia function in algae and humans, and *POLR2G* encodes the RNA polymerase II subunit G [38,39]. Moreover, *PARS2* encodes a putative member of the class II family of aminoacyl-tRNA synthetases and those with mutations in *PARS2* could have Alpers syndrome [40].

The second member of the trio TF, GCM1, is a well-known TF involved in the regulation of expression of placental growth factor (PGF) and other placenta-specific genes [41]. Within the top trio network, GCM1 targets 20 genes (*AATF*, *ADAM33*, *ALS2CL*, *CHRNA3*, *CLSTN2*, *CTTNBP2*, *DRC1*, *EPC2*, *FAM134C*, *GUCY1A2*, *ICA1L*, *KIAA1324L*, *KIRREL3*, *LOC100523745*, *LOC100626814*, *LRFN2*, *MAP3K14*, *PLAT*, *PPP6R3*, and *RGL1*). Among them, three (*CLSTN2*, *MAP3K14*, and *KIRREL3*) were involved in developmental process, similar to GCM1.

Finally, GLI2 functions as a transcription regulator in the Hedgehog (Hh) pathway. Sonic Hh (Shh) functions as a conserved morphogen in the development of various organs in metazoans—from *Drosophila* to humans [42]. It has also been reported that GLI2 is required for the proper development of placental labyrinth [43]. Among the 20 target genes of GLI2 (*ADAM33*, *ALS2CL*, *ARHGAP39*, *CHRNA3*, *DAB1*, *ENSSSCG00000027019*, *EPC2*, *GUCY1A2*, *KIAA1324L*, *KIRREL2*, *LOC100155825*, *LOC100515685*, *LRFN2*, *OXNAD1*, *PKM*, *PPFIBP1*, *PPP6R3*, *SMARCD1*, *STAG1*, and *TNS3*), two (*DAB1* and *KIRREL2*) were related to the developmental process. The GCM1 and GLI1 modules shared eight target genes (*ADAM33*, *CHRNA3*, *LRFN2*, *GUCY1A2*, *EPC2*, *PPP6R3*, *ALS2CL*, and *KIAA1324L*).

## 4. Conclusions

This study not only provides a list of chromosomal regions and SNPs associated with economically important traits, but also their candidate associated genes. The information about the SNP markers and chromosomal regions associated with the studied traits could be considered as prior information in a genomic selection model. Additionally, to the best of our knowledge, this is the first study to propose a BF-based regulatory gene network, unlike AWM with *p*-value information reported previously. This co-association regulatory network created using BFAT as a key trait, would facilitate the validation of the genetic understanding of other economically important traits in pigs. These biologically non-similar traits network could be very useful for the development of improved breeding strategies in the future. Further studies are needed to clarify the specific molecular or cellular processes of interaction among the TF trios and their target gene networks predicted to determine economically important traits in pigs.

## Figures and Tables

**Table 1:** Variance components and heritability estimated for growth and reproductive traits in Yorkshire pigs.

Trait ^1^	N	Mean	SD	Min.	Max.	$\sigma_{A}^{2}$	$\sigma_{P}^{2}$	$h^{2}$
BFAT (mm)	39,406	13.76	2.96	7.60	23.20	2.93	6.99	0.36
ADG (g)	39,516	609.30	74.48	449.00	952.00	0.12	0.35	0.42
DAYS (days)	39,221	149.3	14.4	112.00	188.00	0.44	1.26	0.35
PCL (%)	39,508	58.14	2.97	49.60	65.80	3.48	8.33	0.42
LTTNB	39,518	11.80	2.95	2	25	0.76	7.20	0.11
LTNBA	39,518	10.65	2.70	1	24	0.73	7.07	0.10
WEI	12,975	4.88	1.40	1	15	0.15	1.86	0.08

^1^ BFAT = backfat thickness; ADG = average daily gain; DAYS = days to 90-Kg body weight; PCL = lean percent; LTTNB = lifetime total number of born; LTNBA = lifetime number of born alive; WEI = weaning to estrous interval.

**Table 2:** Informative 1-Mb genome windows and single nucleotide polymorphisms (SNPs) within windows associated with growth rate (ADG and DAYS) and production (BFAT and PCL) traits in Yorkshire pigs from the GWAS using markers on Illumina PorcineSNP60.

Trait ^1^	SSC_Mb ^2^	GV%	Informative SNP	rs Number	Position (Mb)	Effect	BF ^3^	Region Annotation	Gene Annotation
BFAT	2_162	3.51	ASGA0084103	rs81341288	162.15	0.092	57.2	intronic	*COX8H*, *IFITM2*, *IFITM3*
			ASGA0085784	rs81328276	162.3	0.085	49.58	intronic	*IFITM2*, *IFITM3*
	2_76	2.87	MARC0048160	rs81239450	76.09	−0.076	41.77	intergenic	*GNA11*(dist = 3121), *THOP1*(dist = 278492)
			MARC0030590	rs81224732	76.32	−0.056	28	intergenic	*GNA11*(dist = 227056), *THOP1*(dist = 54557)
	2_0	2.68	ASGA0097367	rs81317307	0.37	0.132	141.12	intergenic	*IRF7*(dist = 63158), *PHLDA2*(dist = 62536)
			ASGA0098481	rs81318741	0.92	0.039	34.24	intergenic	*NAP1L4*(dist = 452192), *FADD* (dist = 644399)
	15_132	2.31	INRA0050241	rs339585634	132.56	0.126	244.75	intergenic	*LOC100738836*(dist = 879831), *ARPC2*(dist = 791438)
	5_65	2.15	ALGA0114229	rs81343150	65.63	0.146	304.09	intergenic	*MFAP5*(dist = 49502), *CD163L1*(dist = 347959)
	14_142	1.13	ALGA0082467	rs80835167	142.22	−0.084	126.1	intergenic	*MCMBP* (dist = 953163), *FGFR2*(dist = 277982
	8_11	1.08	MARC0034108	rs81227701	11.32	0.04	40.69	intergenic	*CD38*(dist = 542438), *QDPR* (dist = 1008577)
	14_4	0.89	ALGA0074404	rs80792287	4.23	0.078	136.21	intergenic	*SYK* (dist = 1355883), *LPL* (dist = 230942)
	16_79	0.93	ALGA0091967	rs81462835	79.94	−0.077	133.5	intergenic	*TNIP1*(dist = 1618306)
ADG	17_17	1.88	INRA0052808	rs342665431	17.55	0.03	298.75	intergenic	*BMP2*(dist = 135846), *HAO1*(dist = 1265868)
	5_93	1.4	DRGA0006163	rs345168974	93.83	0.025	209.22	intergenic	*SOCS2*(dist = 113304), *BTG1*(dist = 1259303)
	10_28	1.1	ALGA0057938	rs81422478	28.94	−0.02	89.32	intergenic	*TNNI1*(dist = 728658), *ADIPOR1*(dist = 328094)
	1_177	1.03	ALGA0006599	rs80799429	177.01	0.015	54.25	intergenic	*SERPINB10*(dist = 1651991), *RNF152*(dist = 58137)
	2_2	0.89	M1GA0002244	rs81362590	28.34	−0.017	103.16	intronic	*CPT1A*
DAYS	17_17	2.22	INRA0052808	rs342665431	17.55	−0.065	764.87	intergenic	*BMP2*(dist = 135846), *HAO1*(dist = 1265868)
	5_93	1.12	DRGA0006163	rs345168974	93.83	−0.032	111.06	intergenic	*SOCS2*(dist = 113304), *BTG1*(dist = 1259303)
PCL	2_162	5.87	ASGA0085784	rs81328276	162.3	−0.212	154	intronic	*IFITM2*, *IFITM3*
			ASGA0084103	rs81341288	162.15	−0.13	61.09	intronic	*COX8H*, *IFITM2*, *IFITM3*
	2_0	4.48	ASGA0097367	rs81317307	0.37	−0.287	1268.4	intergenic	*IRF7*(dist = 63158), *PHLDA2*(dist = 62536)
	2_76	1.49	MARC0048160	rs81239450	76.09	0.053	25.2	intergenic	*GNA11*(dist = 3121), *THOP1*(dist = 278492)
	15_132	1.37	INRA0050241	rs339585634	132.56	−0.078	77.91	intergenic	*LOC100738836*(dist = 879831), *ARPC2*(dist = 791438)
	5_65	1.28	ALGA0114229	rs81343150	65.64	−0.362	214.29	intergenic	*MFAP5*(dist = 49502), *CD163L1*(dist = 347959)
	6_157	1.15	M1GA0009131	rs81394508	157.39	−0.175	221.8	intergenic	*GUCA2B* (dist = 903014), *MIR30C*-1(dist = 85847)
	14_4	1.06	ALGA0074404	rs80792287	4.23	−0.121	281.92	intergenic	*SYK* (dist = 1355883), *LPL* (dist = 230942)
	7_18	0.99	MARC0003814	rs80894864	18.13	0.15	205.05	intergenic	*ID4*(dist = 1948801), *PRL* (dist = 284338)
	4_42	0.86	INRA0013856	rs337241703	42.84	0.131	110.57	intronic	*CPQ*

^1^ BFAT = backfat thickness; ADG = average daily gain; DAYS = days to 90-kg body weight; PCL = lean percent; ^2^ SSC_Mb = Sus scrofa chromosome_megabase-pair; ^3^ Bayse factor.

**Table 3:** Informative 1-Mb genome windows and single nucleotide polymorphism (SNPs) within windows associated with reproduction traits in Yorkshire pigs from the GWAS using markers on Illumina PorcineSNP60.

Trait ^1^	SSC_Mb ^2^	GV%	Informative SNP	rs Number	Position (Mb)	Effect	BF ^3^	Region Annotation	Gene Annotation
LTTNB	16_78	1.45	ASGA0074339	rs81462568	78.56	0.057	79.31	iIntergenic	TNIP1 (dist = 236491)
	1_9	1.13	DIAS0003564	rs80972878	9.86	−0.044	53.1	iIntergenic	SOD2 (dist = 373282), TAGAP (dist = 183449)
	16_44	1.1	MARC0073405	rs81259195	44.88	0.031	34.12	intergenic	RGS7BP (dist = 1268524)
			ASGA0073217	rs81459064	44.83	0.02	20.38	intergenic	RGS7BP (dist = 1315507)
LTNBA	1_177	1.38	ASGA0004992	rs80843328	177.74	0.034	23	intergenic	RNF152 (dist = 679283), MC4R (dist = 808927)
WEI	12_57	1.96	ASGA0092942	rs81311789	57.41	−0.024	100.2	intergenic	NTN1 (dist = 349904), GLP2R (dist = 32195)

^1^ LTTNB = lifetime total number of born; LTNBA = lifetime number of born alive; WEI = weaning to estrus interval; ^2^ SSC_Mb = Sus scrofa chromosome_megabase-pair; ^3^ Bayse factor.
